# Advances in Neurological Pain Management: Bridging Scientific Innovations and Clinical Practice

**DOI:** 10.7759/cureus.80447

**Published:** 2025-03-12

**Authors:** Subodh Raj Khadka, Pavani Karani, Neha Gogineni, Indiravati Vaddadi, Chet Raj Awasthi, Nicole Gajowski, UFN Rizwanullah, Christian Aponte Hernández, Hafiz Muhammad Irfan Razzaq, Shehriyar

**Affiliations:** 1 Medicine, Croydon University Hospital, London, GBR; 2 Clinical Research, Oxford College, Toronto, CAN; 3 Family Medicine, University Hospitals Cleveland Medical Center, Toronto, CAN; 4 Critical Care Medicine, Christian Medical College, Vellore, IND; 5 General Medicine, Watford General Hospital, Watford, GBR; 6 Internal Medicine, Avalon University School of Medicine, Willemstad, CUW; 7 Internal Medicine, Hayatabad Medical Complex Peshawar, Peshawar, PAK; 8 Medicine and Surgery, University of Medicine and Health Sciences, Basseterre, KNA; 9 Neurosurgery, Punjab Institute of Neurosciences (PINS), Lahore, PAK

**Keywords:** neurological pain, neuromodulation, pain management, personalized therapy, regenerative medicine

## Abstract

Neuronal pain, including neuropathic pain, migraines, and chronic pain syndromes, presents a significant global health challenge. This literature review covers studies conducted until 2024 using major databases, including PubMed and Google Scholar, with the search terms "Neuropathic Pain/therapy" OR "Chronic Pain/therapy" OR "Pain Management/methods" OR "Neuromodulation/methods" OR "Spinal Cord Stimulation" OR "Deep Brain Stimulation" OR "Transcranial Magnetic Stimulation" OR "Transcranial Direct Current Stimulation" OR "Nav1.7 Voltage-Gated Sodium Channel" OR "Biologics/pharmacology" OR "Drug Delivery Systems/methods" OR "Regenerative Medicine/methods" OR "Stem Cell Transplantation/methods" OR "Platelet-Rich Plasma/therapeutic use" OR "Tissue Engineering/methods" OR "Biomarkers/metabolism" OR "Machine Learning" OR "Precision Medicine."

This review explores contemporary advancements in neurological pain therapy, emphasizing analytical studies that translate into clinical applications. The research foundation is built on modern literature examining pain mechanisms, pharmaceutical innovations, neuromodulation strategies, personalized pain management, and regenerative medicine.

Notable advancements include neuroinflammation research, molecular and genetic pain factor discoveries, and the development of selective Nav1.7 inhibitors, biologics, and advanced drug delivery systems. Neuromodulation techniques, both invasive (e.g., deep brain stimulation (DBS), spinal cord stimulation (SCS)) and noninvasive (e.g., transcranial direct current stimulation (tDCS), transcranial magnetic stimulation (TMS)), play a crucial role in pain modulation.

Regenerative approaches, including stem cell therapy, platelet-rich plasma (PRP), and tissue engineering, offer promising solutions for tissue repair and symptom relief. Additionally, genomic data, biomarkers, and machine learning enhance precision in pain management. Ethical considerations regarding treatment accessibility and opioid alternatives remain critical, particularly for Hispanic Americans facing language barriers in programs like Optum. Selective serotonin reuptake inhibitors (SSRIs) continue to be widely used in mental health treatment. In conclusion, the convergence of translational research, innovative therapies, and personalized medicine marks a transformative era in neurological pain management, improving patient outcomes and quality of life.

## Introduction and background

The problems associated with neurological pain, including neuropathic pain and migraine, alongside chronic pain syndromes, maintain their status as important clinical problems worldwide. Since neurological pain affects millions of people, it diminishes life quality and generates significant financial and social impacts. The advancement of medical science fails to deliver sufficient pain management to numerous patients who suffer from ongoing discomfort coupled with permanent disability. Researchers have dedicated their studies more and more to uncovering the intricate systems behind neurological pain [1].

Research on molecular genetics and neurophysiology has built a better knowledge about how pain information develops and stays active [2]. Neuroinflammatory processes combined with ion channel abnormalities and modifications of synaptic plasticity now provide researchers with better ways to develop new treatment options.

Research organizations have worked together to convert fundamental discoveries from scientific investigations into useful medical procedures. The movement from laboratory testing to clinical practice includes the establishment of new medical products as well as neuromodulation procedures and regenerative medicine therapies [3]. New pain management approaches emerge from innovations, which include targeted drug delivery systems and biologics together with noninvasive neuromodulation approaches.

This paper summarizes novel advancements in neurological pain treatment strategies that bridge scientific breakthroughs to clinical patient care. The paper analyzes modern discoveries in pain science along with therapeutic progress and translational analysis to explain recent developments in neurological pain medicine.

## Review

Mechanisms of neurological pain

Neuropathic pain is characterized by complex pathophysiological processes that involve neuroinflammation, molecular alterations, and genetic factors. Neuroinflammation, a critical component in the development and maintenance of neuropathic pain, is driven by the activation of glial cells and the release of pro-inflammatory cytokines. Elevated levels of these cytokines contribute to heightened pain sensitivity and are often linked to mood disorders [4]. Specialized pro-resolving mediators (SPMs), such as resolvin D1 (RvD1), have emerged as key regulators in resolving inflammation and pain. Recent studies suggest that neuromodulation techniques can enhance the production of SPMs, facilitating pain relief [5].

Molecular and genetic factors also play a significant role in neuropathic pain. Ion channels, neurotransmitter receptors, and signaling pathways are often dysregulated, leading to abnormal pain processing. Key molecular pathways (Table 1), including transient receptor potential vanilloid 1 (TRPV1), N-methyl-D-aspartate (NMDA) receptors, and voltage-gated sodium channel Nav1.7 (Nav1.7), have been identified as critical contributors to pain signaling and are targeted in therapeutic interventions [5].

**Table 1:** Key molecular pathways in neurological pain TRPV1: transient receptor potential vanilloid 1; NMDA: N-methyl-D-aspartate; Nav1.7: voltage-gated sodium channel Nav1.7 Source [5]

Pathway	Role in pain	Potential target
TRPV1	Nociception	Antagonists
NMDA	Synaptic plasticity	Modulators
Nav1.7	Neuronal excitability	Inhibitors

Neuromodulation techniques in pain management

Various neuromodulation techniques have been developed to address these complex mechanisms, targeting specific neural pathways to modulate pain signals (Table 2).

**Table 2:** Comparison of neuromodulation techniques Source [6-11]

Technique	Mechanism	Clinical application	Advantages
Spinal cord stimulation (SCS)	Inhibits nociceptive transmission	Chronic neuropathic pain	Long-term pain relief
Deep brain stimulation (DBS)	Modulates deep brain pain pathways	Refractory pain conditions	Effective for movement disorders & pain
Transcranial magnetic stimulation (TMS)	Alters cortical excitability	Chronic pain & mood disorders	Noninvasive
Transcranial direct current stimulation (tDCS)	Modifies neuronal activity	Pain modulation & rehabilitation	Low cost & portable

Spinal Cord Stimulation (SCS)

**Figure 1:**
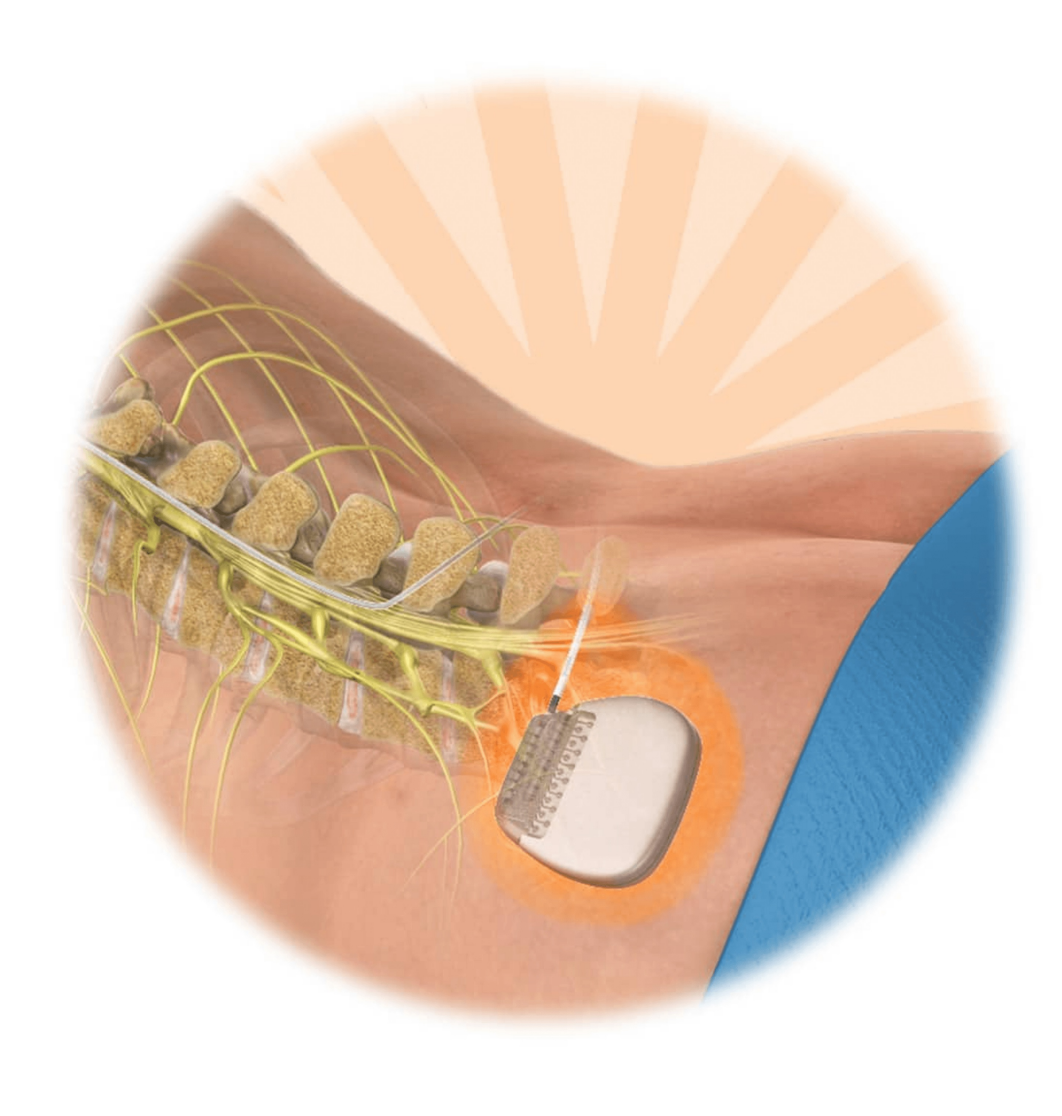
Spinal cord stimulation (SCS) for pain management This figure is taken from the article [12] which is freely accessible under the Creative Commons Attribution-NonCommercial-ShareAlike 4.0 License (http://creativecommons.org/licenses/by/4.0/). This license permits others to modify, adapt, and build upon the work for noncommercial purposes, provided that proper credit is given to the original author, and any new works are distributed under the same terms. The original material remains unaltered

SCS is a well-established interventional treatment for peripheral neuropathic pain. It delivers electrical impulses to the dorsal columns of the spinal cord, inhibiting ascending nociceptive transmission and enhancing descending inhibitory pathways. SCS has shown efficacy in reducing pain intensity and improving the quality of life in patients with chronic pain syndromes [6]. The pulse generator is placed beneath the skin during SCS either in the buttock or abdominal area to reach the spinal cord leads. An electrical impulse transmitting system uses leads to transmit electrical signals for pain signal modification. A patient must undergo a trial procedure to assess stimulation effectiveness before receiving a permanent implant treatment. Figure 1 illustrates this assessment.

Deep Brain Stimulation (DBS)

**Figure 2:**
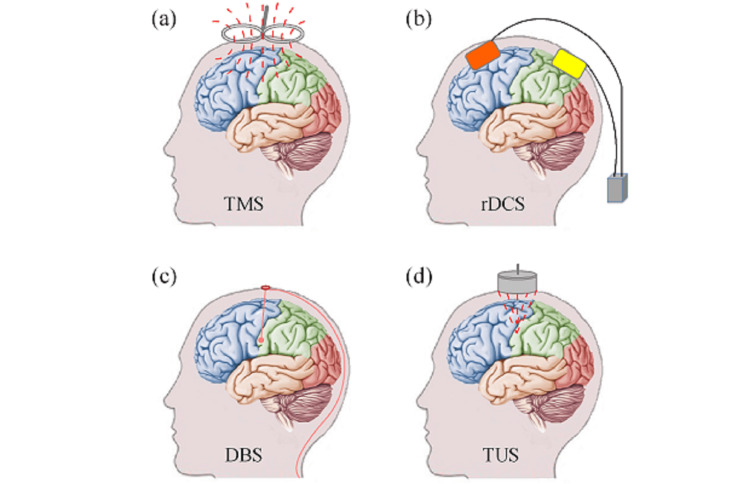
Demonstration of typical neuromodulation techniques: (a) transcranial magnetic stimulation (TMS), (b) transcranial direct current stimulation (tDCS), (c) deep brain stimulation (DBS), and (d) transcranial ultrasound stimulation This figure is taken from the article [13] which is freely accessible under the Creative Commons Attribution-NonCommercial-ShareAlike 4.0 License (http://creativecommons.org/licenses/by/4.0/). This license permits others to modify, adapt, and build upon the work for non-commercial purposes, provided that proper credit is given to the original author

DBS involves the surgical implantation of electrodes into specific deep brain structures. While traditionally used for movement disorders, DBS has shown promise in treating chronic pain conditions (Figure 2, panel c). The technique modulates pain pathways at the thalamic and brainstem levels, offering relief for patients with refractory pain [7,8].

Transcranial Magnetic Stimulation (TMS)

TMS is a noninvasive neuromodulation method that uses magnetic fields to alter cortical excitability (Figure 2, panel a). It is used for treating chronic pain and mood disorders. Studies have shown moderate success rates, with variations depending on stimulation parameters and patient selection [9]. Repetitive TMS (rTMS) applied over the primary motor cortex has been shown to induce long-term analgesic effects by modulating corticospinal and thalamocortical pathways [10].

Transcranial Direct Current Stimulation (tDCS)

tDCS applies low-intensity electrical currents to the scalp to modulate neuronal activity. It is a promising low-cost alternative for chronic pain management, with research highlighting its ability to alter cortical excitability and enhance neuroplasticity (Figure 2, panel b). Studies suggest that combining tDCS with physical therapy can improve functional outcomes and reduce pain in patients with chronic neuropathic pain [11].

While noninvasive methods have limitations in spatial resolution and depth of penetration compared to invasive techniques, recent advancements like focused ultrasound and temporal interference have improved precision and efficacy [8]. These innovations hold promise for expanding the therapeutic applications of noninvasive neuromodulation.

Transcranial Ultrasound Stimulation (TUS)

TUS functions as a nonsurgical brain stimulation method that generates controlled ultrasound energy to adjust neural signaling. Through the unbroken scalp and skull, TUS sends pulse ultrasound waves to the brain destination, wherein it functions as an enhancer or suppressor to neural activity (Figure 2, panel d). Mechanical waves categorized as ultrasound operate above the frequency range audible to humans (more than 20 kHz). The TUS system maintains a basic design that makes its cost comparable to TMS. Standard TUS equipment includes a signal generator combined with a radio frequency amplifier and a piezoelectric transducer, which transforms electrical signal sequences into ultrasonic waves. Focused ultrasound in TUS operates with the ability to focus within millimeters (mm) and reach beyond two centimeters (cm) penetration depths, whereas TMS and tDCS use focused ultrasound, but TUS remains noninvasive like DBS. TUS has received substantial research interest during the past few years because of its numerous beneficial attributes. Tests performed on animals such as rats, rabbits, sheep, and monkeys show that TUS exposure causes no detectable tissue or behavioral damage to brain structures. TUS was successful in treating animal models for diseases, including Alzheimer's disease and epilepsy, while it also enhanced human somatosensory cortex activity and sensory discrimination ability [14].

Advances in pharmacological therapies

Pharmacological interventions remain a cornerstone in the management of neurological pain, with recent advancements focusing on improving efficacy, minimizing side effects, and targeting underlying mechanisms more precisely. Recent years have witnessed the development of novel analgesics aimed at specific pain pathways. One notable advancement is the introduction of selective Nav1.7 sodium channel inhibitors, which target neuronal excitability without affecting other sodium channels, thus reducing the risk of side effects [15]. Another promising class includes cannabinoid receptor agonists, which have shown potential in managing neuropathic pain through the modulation of endocannabinoid signaling [16]. Additionally, N-type calcium channel blockers, such as ziconotide, have demonstrated efficacy in treating severe chronic pain by inhibiting neurotransmitter release in pain pathways [17].

Targeted drug delivery systems have significantly advanced, enabling localized and sustained drug release, thus enhancing therapeutic outcomes and minimizing systemic toxicity. Liposomal formulations and nanoparticle-based carriers have been explored for delivering analgesics directly to affected tissues [18]. Intrathecal drug delivery systems (IDDS) have also gained prominence, particularly for administering opioids and baclofen directly into the cerebrospinal fluid, offering effective pain relief at lower doses [19]. Additionally, gene therapy approaches using viral vectors to deliver antinociceptive genes to specific neural tissues are being investigated as a long-term solution for chronic pain management [20].

Biologics and monoclonal antibodies have emerged as effective therapies for certain types of neurological pain. Monoclonal antibodies targeting nerve growth factor (NGF), such as tanezumab, have demonstrated significant efficacy in reducing pain associated with osteoarthritis and chronic lower back pain [21]. Similarly, calcitonin gene-related peptide (CGRP) monoclonal antibodies, like erenumab and fremanezumab, have been successfully used in migraine prophylaxis by blocking CGRP signaling, a key pathway in migraine pathophysiology [22]. These biologics offer targeted pain relief with fewer side effects compared to traditional analgesics. These pharmacological advancements reflect a shift toward personalized and mechanism-based pain management strategies, promising improved outcomes for patients with complex neurological pain conditions.

Regenerative medicine in pain management and personalized pain management

Advancements in regenerative medicine and personalized approaches have significantly transformed neurological pain management. These strategies focus on restoring damaged tissues and tailoring treatments to individual patient profiles, leading to more effective and sustainable pain relief (Table 3).

**Table 3:** Emerging therapies in neurological pain management Source [21-33]

Therapy	Mechanism	Clinical evidence	Future potential
Stem cell therapy	Tissue regeneration & immune modulation	Promising results in trials	Long-term pain relief potential
Platelet-rich plasma (PRP)	Enhances healing & reduces inflammation	Effective in musculoskeletal pain	Expanding clinical applications
Targeted drug delivery	Site-specific pain relief	Advanced formulations in trials	Precision medicine integration
AI-based pain prediction	Personalized pain treatment	Data-driven models improving diagnostics	Real-time clinical adaptation

Stem cell therapy has emerged as a promising approach for managing chronic neurological pain. Mesenchymal stem cells (MSCs), due to their anti-inflammatory and regenerative properties, have been extensively studied for treating neuropathic pain [23]. MSCs can modulate immune responses, reduce neuroinflammation, and promote tissue repair, leading to pain relief [24]. Clinical trials have demonstrated the potential of stem cell therapy in treating conditions like spinal cord injuries and peripheral neuropathies, though challenges such as cell survival and differentiation remain [25].

Platelet-rich plasma (PRP) therapy involves the injection of concentrated platelets to promote healing and reduce inflammation. PRP is rich in growth factors that stimulate tissue repair and modulate pain pathways [26]. It has been successfully used in treating musculoskeletal pain, including osteoarthritis and tendinopathies [27]. Emerging evidence suggests its potential in neuropathic pain management by enhancing nerve regeneration and reducing neuroinflammation [28].

Tissue engineering combines biomaterials, cells, and bioactive molecules to restore damaged tissues. In pain management, engineered scaffolds seeded with stem cells or growth factors have shown promise in repairing nerve injuries and intervertebral disc degeneration [29]. Hydrogels and nanofibrous scaffolds are particularly effective in supporting cell growth and delivering therapeutic agents directly to the injury site [30]. Personalized pain management leverages genomic data, biomarkers, and advanced computational tools to tailor treatments to individual patients, enhancing efficacy and reducing adverse effects. Genomic profiling and biomarker identification have enabled the development of precision medicine strategies in pain management. Genetic variations in pain-related genes, such as SCN9A and OPRM1, influence pain sensitivity and treatment responses [31]. Biomarkers like cytokine levels and neuroinflammatory markers help predict treatment outcomes and guide therapeutic decisions [32].

Machine learning (ML) algorithms have been increasingly applied in pain prediction and management. These tools analyze large datasets to identify patterns and predict treatment outcomes, enabling more personalized interventions [33]. ML has been used to develop predictive models for chronic pain development, assess treatment efficacy, and optimize pain management strategies [34]. Patient-centered care models emphasize individualized treatment plans that consider patients' preferences, needs, and values. Integrating multidisciplinary teams and incorporating patient feedback into care plans have been shown to improve treatment adherence and outcomes [35]. These models promote shared decision-making and holistic approaches, addressing physical, emotional, and psychological aspects of pain.

Clinical trials of emerging therapies

The transference of laboratory breakdowns into medical treatments depends fundamentally on clinical trial operations. The field of neuromodulation devices, along with new pharmacological agents and regenerative treatments, has received significant trial assessments (Table 4) by Arora and Baldi [3], which presented variable results for both safety and effectiveness. The application of pain research to the clinic has ongoing obstacles from pain system intricacy, patients' irregular reactions, and trials' moral boundaries. A crucial challenge exists for researchers to connect preclinical discoveries to their application in clinical practice [36]. The research frontier should unite multiple biological data methods with custom treatment methods alongside nurse-scientist research cooperation to achieve better results. Patient-centered outcomes combined with AI technology help speed up the process of using research findings in medical practice, according to Wiens et al. [37].

**Table 4:** This table summarizes different neuromodulation techniques, including deep brain stimulation (DBS), spinal cord stimulation (SCS), motor cortex stimulation (MCS), transcranial magnetic stimulation (TMS), and transcranial direct current stimulation (tDCS) RCT: randomized controlled trial

Technique	Study design	Sample size	Condition	Outcomes	Adverse effects
DBS	RCT	10	Central post-stroke pain (CPSP)	Significant pain reduction in 70% of patients [8]	Infection (5%), Lead migration (3%)
SCS	Cohort study	30	Neuropathic pain	50% pain reduction in 80% of patients [6]	Device failure (7%), discomfort (5%)
MCS	RCT	18	Trigeminal neuralgia, CPSP	39% of patients had significant pain relief [21]	Seizures (3%), lead dislocation (4%)
TMS	Meta-analysis	25 trials	Chronic pain	Moderate pain reduction (45%) [12]	Headache (10%), dizziness (5%)
tDCS	Systematic review	15 studies	Neuropathic pain	Mild-to-moderate pain relief (35%) [11]	Skin irritation (2%)

Ethical and societal considerations

The crucial ethical matter is to achieve fair access for patients to receive advanced pain management treatments. Medical treatments, including biologics together with neuromodulation devices, face accessibility problems due to high price levels [38]. Social differences create additional barriers that cause unequal treatment results to spread between different groups of people [39]. Healthcare policies combined with programs that subsidize care must be established to guarantee that medical progress benefits all patients.

Among the diverse patient groups, those who are elderly or children, together with people who have cognitive disabilities, experience distinct hurdles when managing their pain. Such populations generally receive insufficient care because healthcare professionals find it hard to evaluate their pain symptoms while also needing to balance treatment risks [40]. Pain management practices should base their approaches on cultural sensitivity while employing customized evaluation tools to eliminate inequality disparities [41]. The understanding of vulnerable patient requirements leads researchers to create better pain management methods that are also safer for patients.

Ethical practices in pain management need to develop because the opioid crisis demonstrates the necessity of minimizing dependency risks and treatment abuse. The excessive use of opioids for long-term pain treatment has created extensive addiction problems together with negative social consequences throughout society [42]. Basing pain treatment on multiple therapeutic methods through nonopioid medications together with physical medicine and behavioral treatments serve as important strategies for reducing opioid medications [38]. Knowledge about opioid risks and benefits needs to be taught to healthcare providers and patients to achieve safer pain treatment methods.

## Conclusions

Recent advancements in neurological pain management have significantly enhanced our understanding of pain mechanisms, enabling the development of more effective therapies. Innovations in pharmacology, including selective ion channel blockers and biologics, have improved the specificity of treatments while reducing adverse effects. Neuromodulation techniques, such as SCS, DBS, and TMS, have shown efficacy in modulating pain pathways, providing relief to patients with chronic and refractory pain conditions. Additionally, regenerative medicine approaches, including stem cell therapy and PRP, have introduced promising methods for repairing damaged tissues and alleviating pain.

Despite these advancements, challenges remain in ensuring widespread access to innovative therapies, particularly for vulnerable populations. Ethical considerations, such as equitable distribution of treatments and addressing opioid dependency, are crucial in shaping future pain management policies. The integration of personalized approaches, leveraging genomic data and machine learning, will further optimize treatment outcomes. Continued research and collaborative efforts between scientists and clinicians will play a vital role in advancing neurological pain management, improving patient care, and enhancing quality of life.
